# 

**Published:** 1914-12

**Authors:** 


					﻿PUBLISHED MONTHLY.	ATLANTA	SUBSCRIPTION $1.00
JOURNAL-RECOg^f
OF MIDK INI^
1 Atlanta Medical and Surgical Journal, Established 1855. )fl„i
and Southern Medical Record, Established 1870.	| v I SI I Cui
' ........,.^....r=== t-
Vol. LXI.	Atlanta, Ga., December, 1914.	No. 9
				

